# “An exploratory randomized, double-blind, placebo- controlled trial protocol on combined Cognitive Behavioral Therapy and adjunct *Brahmi* (Bacopa monnieri) tablets in Premenstrual Syndrome management”

**DOI:** 10.3389/fmed.2025.1657004

**Published:** 2025-09-16

**Authors:** Shahina Khan, Hemavathi Shivapura Krishnarajabhatt, Cinu Mithra Nisa Sushilal, Parvathy Unnikrishnan

**Affiliations:** Department of Stri Roga and Prasuti Tantra (Gynecology and Obstetrics), Amrita School of Ayurveda, Amrita Vishwa Vidyapeetham, Amritapuri, Kollam, India

**Keywords:** Cognitive Behavioral Therapy, Bacopa monnieri, Ayurveda, clinical trial, integrative medicine, Premenstrual Syndrome

## Abstract

Premenstrual syndrome (PMS) is a widespread and under-recognized disorder, characterized by cyclical recurrence of physical, psychological, and behavioral symptoms during the luteal phase, significantly impacting the daily functioning of women. Cognitive Behavioral Therapy (CBT) is an established non-pharmacological strategy for PMS, while *Brahmi* (Bacopa monnieri), a classical Ayurvedic herb, has traditionally been used to enhance cognition, reduce anxiety, and support emotional balance. This exploratory study evaluates whether combining *Brahmi* with CBT offers additional benefit compared to CBT alone. It is a double-blind, randomized, placebo-controlled exploratory clinical trial (CTRI/2025/02/081239) conducted at the Amrita School of Ayurveda, Kollam, Kerala, with sponsorship from the Central Council for Research in Ayurveda Sciences (CCRAS). Forty-six women aged 18–24 years, diagnosed with moderate to severe PMS using the Premenstrual Symptoms Screening Tool (PSST) and the Daily Record of Severity of Problems (DRSP), will be recruited and randomly assigned to receive either CBT with *Brahmi* tablets or CBT with placebo tablets. The primary outcomes include reduction in PMS severity, assessed by DRSP, and improvement in quality of life measured with the Quality of Life Enjoyment and Satisfaction Questionnaire (Q-LES-Q SF). Secondary outcome is reduction in functional disability measured using the Sheehan Disability Scale. Assessments will be conducted at baseline, during treatment, and at follow-up. Statistical analysis will be carried out using repeated measures ANOVA with Bonferroni-adjusted post hoc tests, significance set at *p* < 0.05, and effect sizes reported. A limitation of this design is that it cannot disentangle the individual effects of *Brahmi* from CBT, allowing conclusions only on the combined intervention. The study aims to generate preliminary exploratory evidence for a safe, integrative, and accessible approach to PMS management in young women.

## 1 Introduction

### 1.1 Background

According to the American College of Obstetricians and Gynacologists (ACOG), many women experience physical and emotional changes in the days preceding menstruation. When these symptoms occur cyclically and significantly interfere with a woman’s normal life, they are classified as Premenstrual Syndrome (PMS) (1). In India, reported prevalence estimates of PMS vary widely, ranging from 14.3% to 74.4% (2). Aside from mild forms, PMS can lead to significant disruptions in daily life, contributing to emotional distress, interpersonal challenges, sexual dysfunction, and a range of physical and psychological health problems (3).

If left untreated, PMS can develop into Pre-Menstrual Dysphoric Disorder (PMDD), a more severe psychiatric condition characterized by extreme mood changes, irritability, and functional impairment, including suicidal ideation. PMDD is recognized as a depressive disorder in the DSM-5 (Diagnostic and Statistical Manual of Mental Disorders, 5th Edition) with a prevalence of 3%–8% (4). It is recognized in the International Classification of Diseases (ICD), listed in ICD-10 as “Premenstrual Tension Syndrome” (N94.3) and in ICD-11 as “Premenstrual Dysphoric Disorder” (5), highlighting it as a distinct and clinically significant entity, separate from PMS. Its diagnosis requires symptom tracking over two consecutive menstrual cycles. Therefore, early identification and effective intervention for PMS are critical to prevent progression to PMDD.

### 1.2 Literature review

A comprehensive literature review was undertaken to explore both modern and Ayurvedic perspectives on Premenstrual Syndrome (PMS) and the potential role of *Brahmi* (Bacopa monnieri). Electronic databases including PubMed, ScienceDirect, and Web of Science were searched using keywords such as *“Premenstrual Syndrome,” “Cognitive Behavioral Therapy,” “Ayurveda,” “Bacopa monnieri,” “Integrative medicine,”and “Clincal Trial*.” Classical Ayurvedic texts (*Charaka Samhita, Sushruta Samhita, Ashtanga Hridaya)* were reviewed for symptomatology analogous to PMS and descriptions of *Brahmi*. Relevant studies, clinical trials, and reviews published in English were included, with emphasis on those addressing psychological, and neuroendocrine aspects. References were selected based on their scientific relevance and credibility to ensure a holistic understanding of the topic.

The management of Premenstrual Syndrome (PMS) often requires a multimodal approach, encompassing pharmacological options such as combined oral contraceptives and selective serotonin reuptake inhibitors, along with non-pharmacological strategies including psychological interventions and lifestyle modifications (3). Cognitive Behavioral Therapy (CBT), in particular, is a structured, evidence-based intervention designed to help individuals recognize and modify maladaptive thoughts and behaviors (3). Evidence suggests that CBT can be beneficial in alleviating the emotional and psychological symptoms associated with PMS, thereby contributing to improved quality of life (6, 7).

While there is no direct reference to PMS in classical Ayurvedic texts, symptoms closely resembling PMS have been described under various functional imbalances. Traditional Ayurvedic scholars recognized that physical illnesses can affect mental health and vice versa—an idea consistent with modern psychosomatic medicine (8). Symptoms of *Vata* vitiation—*the principle governing movement and nervous system functions*—include muscle stiffness, headache, fatigue, body ache, insomnia, and sadness (9). *Pitta*—*the principle of metabolism, heat, and transformation*—when aggravated, manifests as anger and confusion (10). *Kapha*—*the principle of structure, stability, and fluid balance*—in excess produces lethargy, excessive sleep, and nausea (11). *Udāvarta*, a pathological state attributed to the obstruction or reverse flow of *Vata*, particularly from suppression of natural urges, presents with abdominal distension, bloating, constipation, heaviness, emotional instability, and mental disturbances (12). These descriptions correspond closely to the symptomatology of PMS. Furthermore, in more severe cases like PMDD, additional symptoms of abdominal distention and cramps (13), emotional agitation, crying spells, and abnormal behavior have also been described (14). In Ayurveda, *Unmada* is described as a spectrum of mental disturbances characterized by disorganized thought, emotional dysregulation, and impaired social functioning, which further parallels the severe psychological presentations of PMDD.

### 1.3 Ayurvedic perspective

In Ayurveda, *Brahmi* (Bacopa monnieri) is regarded as a *Medhya Rasayana*, a rejuvenating herb for the mind and nervous system. Classical texts describe it as a tonic that nurtures memory, reduces stress, and promotes emotional balance. It has long been used to calm the mind and strengthen mental resilience, qualities that are particularly relevant when addressing the emotional fluctuations seen in PMS (15).

### 1.4 Modern pharmacological perspective

Modern research supports these traditional insights by showing that *Brahmi* acts on several important brain chemicals, including serotonin, dopamine, Gamma-Aminobutyric Acid (GABA), and acetylcholine—all of which are linked to mood regulation and PMS (16, 17). It also contains natural compounds like flavonoids and saponins that protect the brain from oxidative stress, reduce inflammation, and improve communication between nerve cells. These effects are thought to work through multiple pathways, such as cholinergic (18), dopaminergic (19), serotonergic (20), and GABAergic systems (18).

Interestingly, *Brahmi* also benefits gut health. Since most of the body’s serotonin is produced in the gut, disturbances there can affect both mood and inflammation. With its prebiotic properties, *Brahmi* helps restore a healthier gut environment, supporting the gut–brain connection and contributing to emotional stability (21, 22).

Together, these traditional and modern perspectives suggest that *Brahmi* may play an important role as a supportive therapy for PMS, especially in easing mood-related and neuroendocrine symptoms.

### 1.5 Need for the trial

Premenstrual Syndrome (PMS), if unaddressed, may progress to Premenstrual Dysphoric Disorder (PMDD), affecting mental health and daily life. Early diagnosis and timely care are vital. This trial explores whether combining Cognitive Behavioral Therapy (CBT) with oral *Brahmi* (Bacopa monnieri) tablets offers added benefit over CBT alone in managing moderate to severe PMS.

### 1.6 Research hypothesis

This exploratory study hypothesizes that combining Cognitive Behavioral Therapy (CBT) with oral intake of *Brahmi* (Bacopa monnieri) 500 mg once daily for two menstrual cycles may offer additional improvement in Premenstrual Syndrome (PMS) symptoms compared to CBT alone, based on Brahmi’s potential neurocognitive, emotional, and gut-brain modulatory properties.

## 2 Study objectives

### 2.1 Outcome measures

#### 2.1.1 Exploratory primary objectives

- To explore the potential effect of Cognitive Behavioral Therapy (CBT) with versus without oral administration of 500 mg Brahmi tablet once daily on the severity of premenstrual symptoms, as assessed by the Daily Record of Severity of Problems (DRSP) (23).
- To explore the impact of the intervention on quality of life, as assessed using the Quality-of-Life Enjoyment and Satisfaction Questionnaire Short Form (Q-LES-Q SF 12) (24).

#### 2.1.2 Exploratory secondary objective

- To explore the effect of the intervention on functional impairment during PMS-related menstruation, as assessed by the Sheehan Disability Scale (SDS) (25).

#### 2.1.3 Rationale for DRSP

The DRSP is a validated daily symptom diary designed to capture both physical and affective premenstrual symptoms according to DSM-5 criteria. Its prospective format minimizes recall bias and allows precise tracking of symptom fluctuations, making it appropriate for evaluating the efficacy of interventions in PMS.

## 3 Methods

### 3.1 Trial design

This study is a double-blind, placebo-controlled, randomized exploratory clinical trial designed to generate preliminary observations on feasibility, safety, and potential signals of *Brahmi* (Bacopa monnieri) tablets as an adjunct to Cognitive Behavioral Therapy (CBT) in managing Premenstrual Syndrome (PMS). Eligible participants will be randomly assigned to one of two groups: the trial group (CBT plus Brahmi) or the control group (CBT plus placebo). Randomization will be performed using a fixed block design with a block size of four to ensure balanced group sizes, with the sequence generated by a computer algorithm and maintained by a Medical Officer who is not involved in participant recruitment, intervention delivery, or outcome assessment. Both participants and study personnel involved in delivering CBT, assessing outcomes, and analyzing data will remain blinded to group assignments, ensuring methodological rigor and minimizing potential bias throughout the exploratory trial.

### 3.2 Study settings

The study will be conducted at Amrita Ayurveda Medical College Hospital, located in Clappana, Vallikavu, Kollam, Kerala, India – 690525. This institution is a constituent unit of Amrita Vishwa Vidyapeetham and serves as both a teaching and research hospital.

### 3.3 Eligibility criteria

Participants must give written, informed consent before participating in any study procedures.

#### 3.3.1 Inclusion criteria

- Participants aged 18–24 years will be included to align with international youth definition while ensuring safety, methodological homogeneity, and minimizing confounding factors (26, 27).
- Regular menstruation with a menstrual interval of 25–35 days over the past 6 months.
- Participants scoring below 10 on the Patient Health Questionnaire-9 (PHQ-9) will be included, thereby excluding moderate to severe depression and ensuring the selection of individuals with PMS-related mood symptoms only.
- Diagnosed with moderate to severe PMS through PSST, the diagnosis will be confirmed with screening by the Daily Record of Severity of Problem (DRSP) scale for 2 menstrual cycles.

#### 3.3.2 Exclusion criteria

- Known cases of Hypertension, Thyroid disorders, and Diabetes Mellitus. (Participants with hypertension (28), diabetes mellitus (29), or thyroid dysfunction (30) will be excluded to minimize confounding, as these conditions independently influence cardiovascular, metabolic, hormonal, and mood-regulating pathways that overlap with PMS pathophysiology).
- Under medication for any psychological or psychiatric disorders, substance use.
- Current PMS/PMDD treatment.
- Known case of pelvic pathology.
- Planning to conceive during the study period.
- Hormonal therapy.
- No access to mobile phones and the internet.

### 3.4 Interventions

The study intervention will span two menstrual cycles. Participants in the intervention group will receive one-to-one online Cognitive Behavioral Therapy (CBT) sessions, combined with a 500 mg *Brahmi* (Bacopa monnieri) tablet administered once daily after (31, 32) meals with lukewarm water. (33, 34) The control group will receive identical CBT sessions alongside a 500 mg placebo tablet containing inert excipients (dicalcium phosphate, talc, and starch), also taken once daily after food with lukewarm water. Oral medications will be withheld during the menstrual bleeding period for both groups.

The *Brahmi* and placebo tablets were procured from a GMP-certified manufacturer and underwent quality control and analysis to ensure safety, potency, and consistency. Both *Brahmi* and placebo tablets are film-coated to provide uniform appearance, stability, and ease of administration, with the placebo identical in color, shape, and packaging to maintain the double-blind design of the exploratory study.

Cognitive Behavioral Therapy (CBT) sessions will be conducted by two certified CBT therapists, both of whom hold a Master’s degree in Counseling Psychology and formal certification in CBT from accredited institutions. Each therapist has over 2 years of clinical experience in delivering CBT for stress- and mood-related disorders in outpatient settings.

Sessions will be delivered individually (one-on-one) to allow personalized assessment and guidance, ensuring that the intervention is tailored to each participant’s needs. In the event that one therapist is unavailable, the other will continue the intervention without disruption to maintain consistency.

A total of eight sessions will be delivered over two menstrual cycles, on the 5th, 12th, 19th, and 26th days of each cycle, with each session lasting approximately 45 min to 1 h. The CBT protocol is adapted from a previously published study on PMS and includes:

Protocol for Cognitive Behavioral Therapy sessions adapted from a standardized published study on CBT for PMS (7):

- Following the previous study in Iran, a total of 8 sessions will be conducted. Except for 1*^st^* and last sessions, all sessions include both cognitive and behavioral aspects.
- **Session 1:** The general information on PMS/PMDD and its etiology are presented. While the last session included the instructions for the patients, such as the methods for relapse prevention. Both the cognitive and behavioral strategies were discussed in sessions 2 to 7.
- **Session 2:** Psychological training on the role of thoughts and their relationship with emotions and behavior (cognitive triangle) and psychological training on the relationship between stress and PMS and teaching relaxation techniques.
- **Session 3:** Changing the topic to PMS and psychological training on the interdependence between nutrition, exercise, and PMS.
- **Session 4:** Reconstruction of dysfunctional perceptions and How to integrate exercise into daily life using a motivational program and strategy.
- **Session 5:** Psychological training on certain superstitions about PMS and applying the cognitive strategies learned and what is a balanced diet and how to follow it in daily life.
- **Session 6:** Psychological training on effective thoughts and developing new assessments and Psychological training on the impact of stress-related errors on reasoning.
- **Session 7:** Certain behaviors to improve PMS (use of healthcare, seeking support, and communication) and Training on participating in positive activities in daily life.
- **Session 8:** Instructions for the participants such as the methods of relapse prevention.

### 3.5 Dose selection

The classical texts did not use the metric system; doses were described using traditional units such as *ratti* and *karsha*. According to *Bhāvaprakāśa Nighan.t.u*, the dose of *Brahmi* Choorna ranges from 4 to 8 *ratti*, where 1 *ratti* is approximately 125 mg, corresponding to 500–1,000 mg (35). The *Sharangadhara Sam.hita* mentions a general dose of 1 *Karsha* (≈12 g) (36). For modern clinical application and standardization, the Ayurvedic Pharmacopoeia of India (API) recommends 1–3 g of *Brahmi* Choorna. (37) Considering the intervention duration of 2 months in this study, a lower-end dose of 500 mg per day was selected to ensure safety while remaining within the classical and standardized dose range. This dose aligns with both traditional guidance and contemporary clinical practice, ensuring participant safety without compromising potential efficacy.

### 3.6 Choice of comparator

Cognitive Behavioral Therapy (CBT) has been chosen as the comparator treatment due to its well-documented efficacy in alleviating symptoms of PMS. However, there is evidence that symptom recurrence can occur after discontinuation of CBT. To enhance the durability and breadth of therapeutic effects, this study introduces *Brahmi* as an adjunct therapy. Brahmi’s known cognitive, neurochemical, and gut-regulating effects may provide complementary benefits when used alongside CBT.

In this trial, the intervention group will receive CBT along with a 500 mg *Brahmi* tablet, while the comparator group will receive CBT with a placebo tablet identical in appearance. This design will help to understand the combined effect of CBT and *Brahmi* in enhancing symptom control and functional outcomes.

### 3.7 Intervention modifications

If participant misses their scheduled morning dose of the *Brahmi* tablet or the placebo tablet, the following protocol will be implemented:

- Same-day missed dose: If the participant realizes the missed dose within the same day, they may take the dose in the evening, provided it is at least 6 h after the previous dose.
- Next-day missed dose: If the missed dose is realized the following day, the participant should skip the missed dose and resume the regular dosing schedule at the next scheduled time.

Regarding missed Cognitive Behavioral Therapy (CBT) sessions, if a participant is unable to attend a scheduled session, the following options are available:

- Rescheduling: The session may be rescheduled to the next available day, ensuring that the session occurs within 24 h of the original appointment.
- Preponing: With prior notice, the session may be conducted earlier than the scheduled time, provided it is within the same week.

These flexible scheduling options aim to accommodate participants’ availability and minimize disruption to their treatment plan.

### 3.8 Protocol adherence criteria

#### 3.8.1 Adherence to *Brahmi* intake

- Medication consumption: participants will be considered adherent if they consume at least 80% of the prescribed *Brahmi* tablets during the intervention period.
- Pill count verification: compliance will be objectively monitored by pill counts conducted at each follow-up visit. Any discrepancy will be documented in the case record form.
- Missed doses: participants will be instructed to record any missed doses in a provided diary. Repeated non-adherence (e.g.,> 20% missed doses) will be flagged as protocol deviation.
- Correct usage: intake of the drug must align with provided instructions, i.e., correct dosage schedule and proper storage.

#### 3.8.2 Adherence to CBT participation

- Attendance requirement: participants will be considered adherent if they attend at least six sessions (75%–80%) of the scheduled CBT sessions.
- Session documentation: attendance will be recorded by the therapist/facilitator after each session, and non-attendance will be followed up by reminder calls.
- Engagement in sessions: active participation (completion of exercises, discussions, and assignments) will also be documented to assess qualitative adherence.
- Make-up sessions: if participants miss a session, opportunities for catch-up or supplementary guidance will be offered, ensuring minimal loss of therapeutic continuity.

#### 3.8.3 Combined adherence monitoring

- Overall protocol adherence will be defined as meeting both the minimum thresholds for *Brahmi* intake (≥ 80%) and CBT session attendance (≥ 75%–80%).
- Participants failing to meet either criterion will be considered partial adherents, and this will be documented in adherence logs for sensitivity analysis during final data evaluation

### 3.9 Adherence-improving strategies

To ensure compliance with both the medication and CBT interventions, the following strategies will be employed:

#### 3.9.1 Follow-up sessions

Participants will be called for follow-up sessions during both screening and treatment phases, where the importance of adherence to study protocols will be reinforced.

#### 3.9.2 Medication guidelines

- Clear instructions will be provided regarding dosage schedule, storage conditions, and the necessity of taking tablets intact.
- Procedures for managing a missed dose will be explained.
- Participants will be reminded about the objectives and expected benefits of the intervention.

#### 3.9.3 Pill count monitoring

Pill counts will be conducted and documented at every study visit to objectively assess medication adherence.

#### 3.9.4 CBT session attendance

- Attendance at each CBT session will be documented through session logs and trainer records.
- Missed sessions, along with reasons, will be recorded.
- Therapists will maintain brief notes on participant engagement to ensure fidelity of the intervention.

#### 3.9.5 Adherence reminders

At each visit, participants will be reminded of the significance of adhering to study requirements for reliable outcomes.

#### 3.9.6 Reporting issues

Participants will be encouraged to immediately contact the study center in case of any difficulties with medication intake or CBT participation, including side effects or scheduling challenges.

### 3.10 Rescue medication

In case of nausea or vomiting, *Dhanwantharam Gulika* (a polyherbal tablet, traditionally indicated for gastrointestinal disturbances) will be administered. For diarrhoeal episodes, either *Mustarishtam* (a fermented herbal preparation with Cyperus rotundus, used for digestive disorders) or *Kutaja Arishtam* (containing Holarrhena antidysenterica, widely used for diarrhoeal disorders) will be provided, depending on the clinical presentation. For pain or abdominal cramps, participants will be managed with *Hinguvachadi* Tablet (a classical Ayurvedic formulation for abdominal cramps); if symptoms remain refractory, they will be referred to a modern medical practitioner and may be permitted Paracetamol (up to 500 mg as required). For insomnia or heightened anxiety, non-pharmacological measures such as breathing exercises will be recommended. All formulations will be procured from a GMP-certified manufacturer with Quality of Analysis report and use of any rescue medication will be documented for each participant and considered during analysis. No hormonal or psychotropic rescue medications will be allowed during the study period.

## 4 Outcome measures

### 4.1 Exploratory primary outcomes

• Changes in PMS symptom severity, as measured by the Daily Record of Severity of Problems (DRSP) across two intervention cycles.

• Changes in quality of life, as assessed by the Quality-of-Life Enjoyment and Satisfaction Questionnaire Short Form (Q-LES-Q SF 12) from baseline to post-intervention.

### 4.2 Exploratory secondary outcomes

• Changes in functional impairment, as measured by the Sheehan Disability Scale (SDS) total and domain-specific scores, reflecting work, social, and family functioning.

#### 4.2.1 Participant timeline

Participants enrolled in the study will undergo a structured series of visits spanning multiple menstrual cycles. During the first visit, personal history, socio-demographic information, and detailed menstrual history will be recorded. Participants will also complete the Patient Health Questionnaire-9 (PHQ-9) to assess depressive symptoms and the Premenstrual Symptoms Screening Tool (PSST) to evaluate the presence and severity of PMS. Individuals who meet the criteria for moderate to severe PMS based on the PSST will be considered eligible for further participation. At this stage, informed consent will be obtained. During the second visit, which occurs on the 1st or 2nd day of the subsequent menstrual cycle, the screening process will begin. This is followed by an initial Google Meet session at the same phase of the following cycle to confirm ongoing eligibility and provide further study orientation. The third visit (baseline) will be on 1st to 2nd day of the participant’s menstrual cycle, during which assessments using the Daily Record of Severity of Problems (DRSP), Quality of Life Enjoyment and Satisfaction Questionnaire (Q-LES-Q), and Sheehan Disability Scale (SDS) will be conducted. The intervention begins at the fourth visit (aligned with the 1st to 2nd day of the second menstrual cycle), where participants will be randomly assigned to receive either Cognitive Behavioral Therapy (CBT) combined with *Brahmi* tablets (interventional group) or CBT with placebo tablets (comparator group). CBT sessions will be given on the 5*^th^*, 12*^th^*, 19*^th^* and 26*^th^* day for 2 menstrual cycles with total of eight sessions. A final Google Meet follow-up will be conducted during the 1st to 2nd day of the fifth menstrual cycle to assess outcomes and gather participant feedback.

The schedule of visit chart is mentioned in Table 1. It explains the follow-up visits across four menstrual cycles during the study.

**TABLE 1:** Schedule of visit chart.

Activity/assessment	1^st^ visit (history taking, PSST assessment)	2^nd^ visit (start screening 1^st^ to 2^nd^ day menstrual cycle)	G Meet (1^st^ to 2^nd^ day of next menstrual cycle)	3^rd^ visit Baseline visit (1^st^ to 2^nd^ day of menstrual cycle)	4^th^ visit (1^st^ to 2^nd^ day of 2^nd^ menstrual cycle)	G Meet (1^st^ to 2^nd^ day of 5^th^ menstrual cycle)
Personal history, socio-demographic data, and menstrual history	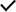					
Patient health questionnaire-PHQ-9 depression scale assessment	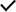					
Pre-Menstrual Syndrome Screening Tool (PSST) assessment	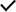					
Informed consent	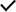					
(DRSP) scale (given only to the participants diagnosed as moderate to severe PMS by PSST) assessment		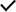	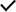	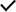	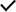	
14 Q-LES-Q assessment				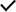	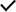	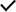
Sheehan Disability Scale (SDS) assessment				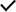	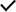	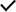
Intervention (CBT with *Brahmi* tablet to Interventional group and CBT with placebo tablet to comparator group. CBT sessions will be given on the 5^th^, 12^th^, 19^th^ and 26^th^ day for 2 menstrual cycles)				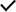	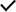	
Feedback on the study and intervention						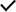

#### 4.2.2 Sample size

The proposed study is a randomized controlled exploratory trial aimed at providing preliminary insights into the comparative outcomes of two independent groups. As formal power calculations are not mandatory for exploratory studies, a two-sample t-test formula for comparing means was applied primarily to provide a rational basis for determining a feasible sample size:


$$n=\frac{2\,s_{p}^{2}\,{\left[Z_{1-\alpha /2}+Z_{1-\beta }\right]}^{2}}{\mu_{d}^{2}}$$

This formula incorporates the pooled variance, the anticipated mean difference between groups, the significance level (α), and statistical power (1−β). Using estimates from published data, the pooled variance (sp^2^) was taken as 2.85, and the expected mean difference (μd) between groups as 1.83. For reference, a two-sided test with a significance level of 0.05 (Z = 1.96) and a nominal power of 90% (Z = 1.28) was considered. Substituting these values yielded a preliminary estimate of approximately 18 participants per group. Allowing for an anticipated dropout rate of 20%, the adjusted number was approximately 23 participants per group, resulting in a total sample size of 46.

#### 4.2.3 Recruitment

To facilitate the recruitment of participants for the study, a comprehensive and multi-faceted awareness campaign will be implemented. This initiative will include two primary components: educational programs and the distribution of informational posters. We will also utilize digital advertisements on various platforms. The purpose of these programs is not only to inform the public about the study but also to raise general awareness about Premenstrual Syndrome (PMS), thereby assisting in the identification of individuals who may be experiencing symptoms. These preliminary initiatives, along with our recruitment channels, have all been approved by the Institutional Ethics Committee. This will be followed by a preliminary screening process, which will be conducted using a Google Form to accurately ascertain the presence of PMS symptoms among potential participants. Furthermore, to uphold ethical standards and avoid any potential for perceived coercion or undue influence, we will not target students only from our university. Individuals identified with such symptoms will be encouraged to seek consultation at the Outpatient Department (OPD) of the Amrita School of Ayurveda, located in Clappana, Kollam, Kerala. Subsequent recruitment into the study will be contingent upon the fulfillment of established inclusion criteria and the completion of the consultation process at the designated study center.

### 4.3 Randomization and allocation sequence generation

Participants who meet the inclusion criteria and complete the initial consultation at the Outpatient Department (OPD) of Amrita School of Ayurveda, Clappana, Kollam, Kerala, will be considered for enrolment. To ensure balanced group sizes and minimize selection bias, participants will be randomly allocated to either the Trial group (CBT + *Brahmi*) or the Control group (CBT + Placebo) using fixed block randomization with a block size of 4. A total of 11 blocks will be formed to accommodate the planned sample of 44, and the remaining two participants will be randomly assigned to any of the existing blocks. The randomization sequence will be generated by the Medical Officer at the Amrita School of Ayurveda. This individual is not involved in participant recruitment, intervention delivery, or outcome assessment, which ensures balanced group sizes throughout enrolment and maintains the internal validity of the study.

### 4.4 Allocation concealment mechanism

A block randomization sequence will be generated by the statistician to ensure balanced allocation between the Brahmi and placebo arms throughout the trial. Based on this sequence, the Medical Officer (who is not involved in assessments) will prepare sequentially numbered, identical, opaque, sealed medication packages. Each package will be coded according to the randomization list but will appear identical in external characteristics, thereby concealing its contents.

At enrolment, participants will receive the next available sequentially numbered package, ensuring strict adherence to the block randomization order. The Principal Investigator, Co-investigators, CBT practitioners, Statistician, and participants will remain blinded to the allocation. The intervention codes will only be revealed after the completion of all post-intervention assessments or earlier in the event of a medical emergency that requires unblinding.

### 4.5 Implementation and blinding

Participant recruitment and enrolment will be conducted solely by the PI at the designated OPD. The Medical Officer will implement the randomization by assigning participants to the trial or control group according to the pre-generated block randomization sequence and dispensing the corresponding medication packages. Cognitive Behavioral Therapy (CBT) sessions will be delivered by certified CBT therapists who remain blinded to participant group assignments. A Co-Investigator will be responsible for assessing the outcomes, and they will also remain blinded to the group assignments. The Statistician will be responsible for data analysis and will also remain blinded until the study is complete.

This study employs a double-blind exploratory design to minimize bias. The following personnel will remain blinded throughout the trial:

- **Participants:** will be unaware of whether they are receiving *Brahmi* or placebo tablets.
- **Principal Investigator (PI):** blinded to group allocation to prevent selection and performance biases.
- **CBT therapists:** administer therapy sessions without knowledge of group assignments to ensure uniformity of intervention delivery.
- **Co-Investigator:** conducts the outcome assessments while blinded to group allocation.
- **Statistician:** conducts data analysis blinded to allocation until study completion to reduce analytical bias.
- **Medical officer:** handles randomization and medication assignment, maintains the confidential coding system, and decodes interventions only after final data analysis or in case of emergency unblinding.

This structured approach ensures rigorous randomization, allocation concealment, and blinding, thereby maintaining methodological integrity and minimizing potential biases throughout the trial.

#### 4.5.1 Emergency unblinding

In emergencies where immediate understanding of the intervention is critical, unblinding may be conducted to prevent harm to the subject. Investigators are urged to consult the Medical Advisor if unblinding is deemed essential.

The Investigator is obligated to uphold the blinding to the greatest extent feasible. The actual allocation must remain confidential from the participant and/or other study personnel, including site personnel or monitors, and there should be no written or verbal disclosure of the code in any related participant papers.

The Investigator is required to document all code violations, along with their justifications, on the relevant CRF page as they arise.

Unblinding should not inherently warrant the cessation of the study drug.

### 4.6 Data collection methods

#### 4.6.1 Pre-screening (demographic data collection)

Before any assessments, participants will fill out a Google Form to provide demographic information and basic eligibility details. The responses will be transcribed into Microsoft Excel for documentation and initial screening purposes.

#### 4.6.2 Initial screening

Participants presenting with PMS symptoms will undergo initial screening using the Premenstrual Symptoms Screening Tool (PSST) and PHQ-9 during a single visit at recruitment (baseline, before Cycle 1). The assessment will be done using paper-pencil in English, and the scores will be recorded in Microsoft Excel.

#### 4.6.3 Pre-intervention observation (screening phase)

During Cycle 1 and Cycle 2, participants will maintain the Daily Record of Severity of Problems (DRSP) daily from Day 1 to the end of the 2nd cycle using paper-pencil in English. A follow-up virtual visit will be conducted on Day 1 or 2 of the 2nd cycle to review progress and clarify doubts. All DRSP scores and notes from the follow-up will be recorded in Microsoft Excel.

#### 4.6.4 Interventional phase

During Cycle 3 and Cycle 4, participants will continue to maintain the DRSP daily from Day 1 to the end of each cycle. Additionally, Sheehan Disability Scale (SDS) and Quality of Life Enjoyment and Satisfaction Questionnaire Short Form (Q-LES-Q-SF) will be assessed on Day 1 or 2 of each cycle using retrospective recall. All scores will be recorded in Microsoft Excel. Assessments will be done using paper-pencil in English.

#### 4.6.5 Documentation

Before enrollment, all participants will be provided with the Informed Consent Form (ICF) and Patient Information Sheet (PIS) in English and Malayalam. Data will be documented using Microsoft Excel with restricted access. Each participant will be assigned a unique study ID code, and no personal identifiers will be included in the main dataset used for analysis. The linkage file containing identifiers will be stored separately under password-protected and encrypted folders accessible only to the Principal Investigator and Co-Investigator. No other trial personnel, including investigators, CBT therapists or Statistician will have access to this master file; they will only handle anonymized case-record forms relevant to their role. Regular backups will be maintained on secure institutional servers to ensure the data integrity. The role-based restricted access ensures confidentiality, prevents unauthorized viewing. These documents will be maintained in paper form, with a digital log as needed.

Table 2 presents the schedule of assessments, detailing the tools, timing, methods, and data recording procedures across the pre-screening, screening, and interventional phases of the study.

**TABLE 2:** Schedule of assessments and data collection methods.

Phase	Assessment tool	Days of assessment	Method	Language	Timeline	Data recording
Pre- screening	Google form (demographics, eligibility)	Before enrollment	Digital → transcribed to Excel	English	Baseline, before Cycle 1	Responses recorded in Microsoft Excel
Initial screening	PSST, PHQ-9	Single visit at recruitment	Paper-pencil	English	Baseline, before Cycle 1	Scores recorded in Microsoft Excel
Pre-intervention observation (screening phase)	Daily Record of Severity of Problems (DRSP)	Daily, Day 1 to end of 2nd cycle	Paper-pencil	English	Cycle 1 and Cycle 2 (screening)	Scores recorded in Microsoft Excel
Pre-intervention observation (screening phase)	Follow-up virtual visit	Day 1 or 2 of 2nd cycle	Virtual	English	After completion of Cycle 2 DRSP	Notes recorded in Microsoft Excel
Interventional phase	Daily Record of Severity of Problems (DRSP)	Daily, Day 1 to end of each cycle	Paper-pencil	English	Cycle 3 and Cycle 4 (intervention)	Scores recorded in Microsoft Excel
Interventional phase	Sheehan Disability Scale (SDS), Q-LES-Q-SF	Day 1 or 2 of each cycle (retrospective recall)	Paper-pencil	English	Cycle 3 and Cycle 4, retrospective assessment of previous cycle	Scores recorded in Microsoft Excel
Documentation	Informed Consent Form (ICF), Patient Information Sheet (PIS)	Before enrollment	Paper	English & Malayalam	Baseline, before Cycle 1	Digital log maintained

#### 4.6.6 Retention

This study informs participants that their involvement is completely voluntary, and they may quit at any time without repercussions. If a participant opts to withdraw for any reason, their decision will be acknowledged. Efforts shall be undertaken to prevent unnecessary dropouts, where feasible. Participants who choose to withdraw will not be subject to any additional data collection or follow-up. This methodology guarantees compliance with ethical norms, honoring participants’ autonomy and rights during the research process.

#### 4.6.7 Data management

Data entry and documentation will be performed both manually, using paper-pencil forms completed by participants, and electronically, using Microsoft Excel. The Principal Investigator (PI) will oversee the process, ensuring the accuracy and completeness of all data and providing clarification to participants if needed. The Co-Investigator will be responsible for entering scores from the paper forms, outcome assessments, and follow-up notes into Excel, maintaining blinding where applicable. All participant data will be organized numerically by participant ID and stored in a secure location to ensure confidentiality while allowing authorized access for study personnel. Only validated scores and documented observations will be included in the analysis. The Statistician, who remains blinded to group allocation, will perform all analyses according to the study protocol, comparing outcomes between the Trial (CBT + *Brahmi*) and Control (CBT + Placebo) groups. Participant confidentiality will be rigorously maintained throughout the study, and all records will be retained for a minimum of 5 years following study completion to allow for verification, audit, or future reference.

### 4.7 Exploratory statistical analysis

Exploratory statistical analysis will primarily explore the potential impact of Cognitive Behavioral Therapy (CBT) combined with either *Brahmi* (Bacopa monnieri) tablets or placebo in participants with moderate to severe Premenstrual Syndrome (PMS). Outcome measures include the Daily Record of Severity of Problems (DRSP), Quality of Life Enjoyment and Satisfaction Questionnaire (Q-LES-Q), and Sheehan Disability Scale (SDS), assessed at three time points: baseline (third visit), during treatment (fourth visit), and post-treatment (fifth cycle follow-up). Analyses will be descriptive, focusing on effect size estimates and 95% confidence intervals to provide insights into the direction and magnitude of observed effects. P values will be presented descriptively without inferential claims, in line with the exploratory nature of the study. Where appropriate, repeated measures approaches such as ANOVA or mixed-effects models may be applied to summarize changes over time and between groups, with emphasis placed on effect size interpretation to generate preliminary signals for future confirmatory research.

### 4.8 Additional analyses

Subgroup analyses may be undertaken to explore potential differential impacts of therapies based on baseline characteristics such as age, severity of PMS symptoms, menstrual cycle regularity, or baseline psychological distress. Adjusted analyses using multivariate models may also be conducted to account for potential confounders (e.g., BMI, diet, baseline stress levels, or prior PMS-related therapies). These will be explicitly categorized as exploratory and interpreted cautiously. Interaction effects between CBT and *Brahmi* will also be examined if the sample size permits. Exploratory analyses may include treatment adherence, participant satisfaction, and patterns in biopsychosocial variables.

### 4.9 Analysis population and missing data

Both intention-to-treat (ITT) and per-protocol analyses will be presented. The ITT analysis will preserve the benefits of randomization and provide insights into real-world effectiveness, while per-protocol analysis will evaluate outcomes in participants with good adherence (≥ 80% intervention completion and no major protocol violations). Missing data will be managed using complete-case analysis as the primary approach. Sensitivity analyses, including last-observation-carried-forward (LOCF), will be performed to assess robustness. Multiple imputation will not be applied, consistent with recommendations for exploratory trials. Mixed-effects models with maximum likelihood estimation may be used for repeated assessments to account for missingness without formal imputation when appropriate. All statistical analyses will be performed using the latest versions of SPSS or R by an independent biostatistician, ensuring objectivity and scientific rigor.

### 4.10 Safety

All adverse events (AEs) and serious adverse events (SAEs) will be systematically documented in the Case Report Form (CRF) using a predefined Adverse Drug Reaction (ADR) page. Each entry will capture the event description, diagnosis (if available), onset and resolution dates, severity (mild, moderate, or severe), action taken with the study drug (none, temporarily interrupted, or permanently withdrawn), outcome (resolved, resolved with sequelae, or not resolved), and the assessed relationship to the study drug (definite, probable, possible, unlikely, not related, or not assessable). The Principal Investigator and Co-Investigator will independently evaluate causality, taking into account the temporal relationship with the study drug, the participant’s clinical course, medical history, and concomitant medications. All adverse events will be followed until resolution or stabilization. Events occurring after a participant exits the study will not be routinely reported unless the investigators consider a reasonable likelihood of relation to the study drug, psychotherapy, or study procedures. This systematic documentation ensures continuous monitoring and prioritization of participant safety throughout the trial

### 4.11 Auditing

This research project will be funded by the Central Council for Research in Ayurvedic Sciences (CCRAS), Ministry of AYUSH. The grant will be utilized strictly as per the approved budget and CCRAS guidelines. All financial records, including bills and receipts, will be maintained in both digital and physical formats and preserved for future audits. Quarterly internal audits will be conducted by the institutional accounts department, and external audits may be carried out by a certified auditor or as directed by CCRAS.

A 6 months progress and financial report will be submitted to CCRAS, including updates on research activities, participant enrollment, preliminary observations, and challenges faced. The financial report will detail fund utilization and be certified by the institutional finance department. Both reports will be submitted in hard and soft copies, and any feedback from CCRAS will be addressed promptly. All procedures will comply with the General Financial Rules (GFR) of the Government of India and institutional norms, ensuring transparency and accountability throughout the study.

## 5 Discussion

Premenstrual syndrome (PMS) represents a significant challenge in women’s health, affecting daily functioning, interpersonal relationships, and overall quality of life. Despite its high prevalence, treatment options often remain limited to symptomatic relief, with pharmacological approaches sometimes associated with side effects or inconsistent acceptability. This underscores the importance of exploring safe, holistic, and patient-centered strategies that not only target symptom reduction but also support long-term wellbeing. Cognitive Behavioral Therapy (CBT) has been consistently reported as an well-established non-pharmacological intervention for mood and anxiety disorders, and emerging evidence highlights its potential relevance in PMS by helping women reframe negative thought patterns, develop adaptive coping skills, and improve emotional regulation. In parallel, *Brahmi* (Bacopa monnieri) holds a respected position in Ayurveda as a *Medhya Rasayana* (nootropic and mind-strengthening herb), traditionally described to enhance memory, concentration, and resilience to stress. Modern pharmacological studies further suggest its role in modulating neurotransmitters such as serotonin and dopamine, reducing oxidative stress, and exerting mild anxiolytic effects, making it worth exploring as a supportive option for PMS-related psychological and somatic symptoms. The novelty of this trial lies in combining these two distinct but complementary approaches. By addressing cognitive patterns through CBT and neurobiological stress responses through *Brahmi*, the study is designed to generate preliminary insights into whether a combined approach may provide broader symptom relief compared to either intervention alone. Importantly, both interventions are non-hormonal, culturally acceptable, and considered safe, which enhances their translational potential in diverse healthcare settings. Methodologically, the trial incorporates several strengths. Participants are screened through standardized tools such as the PSST, PHQ-9, and prospective DRSP diaries, ensuring robust diagnostic confirmation of PMS. The randomized, double-blind design enhances internal validity by minimizing bias, while the use of validated outcome measures including Q-LES-Q-SF and Sheehan Disability Scale ensures that both clinical symptoms and functional impairment are systematically assessed. These choices strengthen the study’s potential to generate initial, reliable observations, even within the constraints of a modest sample size. At a broader level, this work also contributes to the emerging dialogue between traditional systems of medicine and modern psychiatry. While Ayurveda emphasizes balance of body, mind, and environment, CBT provides structured techniques to reframe maladaptive thoughts and behaviors. Integrating the two aligns with the global movement toward holistic, integrative healthcare, where evidence-based traditional interventions are studied with the same rigor as conventional modalities.

## 6 Limitations

This trial is designed as an exploratory study, which in itself places certain boundaries on how the results can be interpreted. By nature, exploratory designs are not meant to provide confirmatory evidence; they mainly help in understanding feasibility and safety. Therefore, the outcomes of this study should be interpreted with caution, as its exploratory character represents the major limitation.

Another limitation comes from the combined intervention model. Since Cognitive Behavioral Therapy (CBT) and *Brahmi* (Bacopa monnieri) will be used together, it will not be possible to know how much each intervention contributes on its own. The results will reflect only the integrated effect of the two, rather than the individual impact of either component.

There are also challenges in aligning the Ayurvedic perspective. PMS and PMDD are not directly identified in classical Ayurvedic texts. Their use in this context is based on correlating symptoms with descriptions found in the literature, rather than on direct reference, which makes the connection more interpretative.

The choice of CBT is supported by earlier studies showing its possible usefulness in PMS and its recognized role in stress- and mood-related conditions. However, it has not yet been included in international guidelines for PMS management. Similarly, while *Brahmi* has a long tradition of use in Ayurveda and growing preclinical support for its role in stress and cognition, it is not a standard treatment in gynecology or psychiatry today.

Taken together, these points underline that this study can only provide preliminary insights into whether a combined CBT–*Brahmi* approach is feasible and potentially helpful. It cannot prove causation or effectiveness. Instead, the findings should be seen as a starting point, paving the way for larger and more definitive studies that can carefully test each component under controlled conditions.

## 7 Conclusion

This exploratory study provides preliminary observations on the potential of an integrative approach combining Cognitive Behavioral Therapy (CBT) and *Brahmi* in the context of Premenstrual Syndrome (PMS). The design does not permit causal interpretation or allow differentiation of the individual contributions of either intervention, and the findings should therefore be regarded as hypothesis-generating rather than definitive. Within these constraints, the study illustrates the feasibility of examining combined psychological and herbal strategies in women’s health and points to areas where further investigation is warranted. Future research, particularly studies with larger samples, diverse populations, and designs that test each intervention separately as well as in combination, will be essential to clarify their roles and establish a more robust evidence base.

## 8 Ethics and dissemination

### 8.1 Research ethics approval

The institutional ethics committee (IEC) meeting was held on 16/01/2025 at Amrita School of Ayurveda. 07 members attended the meeting. Ethics approval received on 19/01/2025.

Ref: IEC.ASA.PJR/06/25.

Letter no. IEC.ASA.PJR/06/25/APPROVAL.

### 8.2 Consent and assent

The principal investigator will present the trial details to the participants. Participants will furthermore receive informational brochures for participants. Participants will subsequently engage in an educated dialogue with the primary investigator. The principal investigator will secure written consent from subjects who agree to partake in the experiment. Patient information papers and informed consent forms are supplied to all participants in the trial. All informed consent documents and patient information sheets have been translated into English and Malayalam.

### 8.3 Confidentiality

All study-related data will be securely maintained at the study site. The participant information will be securely maintained in locked cabinets in restricted access zones. All reports, data collecting, and administrative documents shall be designated by a coded ID number solely to preserve participant confidentiality. All documents, including names or other personal identifiers, including locator forms and informed consent forms, will be retained separately from study data designated by code number. All local databases will be safeguarded with password-protected access systems.

### 8.4 Access to data

Access to the study data will be restricted to the principal investigator, guide, co-guide, external coder and data analyst. All data will be anonymised and stored securely, with electronic files protected by password and physical records kept in locked storage. Only authorized personnel will have access to the identifiable data, and access will be granted solely for the purpose of study-related activities, including data entry, verification, and statistical analysis. Data will not be shared with external parties without prior ethical approval and participant consent. The confidentiality and privacy of all participants will be strictly maintained throughout.

### 8.5 Dissemination policy

#### 8.5.1 Trial results

The investigators and sponsor are committed to the transparent and responsible dissemination of the trial results. Upon completion of the study and analysis of data, the findings will be communicated through multiple channels to ensure that participants, healthcare professionals, the scientific community, and the general public are adequately informed.

Once the study is completed, it will be submitted to peer-reviewed national or international scientific journals, regardless of whether the outcomes are positive, negative, or inconclusive. Additionally, results will be presented at relevant academic conferences and seminars to facilitate knowledge exchange among clinical and research professionals.

Participants who express interest will be informed of the overall study findings in lay language through written summaries or verbal debriefings. The research outcomes may also be registered and shared through publicly accessible clinical trial registries or results databases, following ethical and regulatory requirements.

There are no publication restrictions imposed by the sponsor. However, all publications and public disclosures will undergo review by the principal investigator and ethics committee to ensure accuracy, integrity, and compliance with institutional and ethical standards. Any data shared publicly will be de-identified to protect participant confidentiality.

#### 8.5.2 Authorship

Authorship will be based on the ICMJE criteria, requiring substantial contribution to study design, data acquisition or analysis, manuscript drafting or revision, final approval of the version to be published, and accountability for all aspects of the work. Individuals who do not meet these criteria but contribute meaningfully will be acknowledged with their consent.

The use of professional medical writers is not planned. If engaged, their involvement will be transparently disclosed, and authorship will only be granted if ICMJE criteria are met. Final authorship decisions will rest with the principal investigator in line with ethical and institutional guidelines.
